# Comparison of residual stress distribution between root-analogue implant and threaded cylindrical implant

**DOI:** 10.1186/s12903-025-06412-5

**Published:** 2025-07-03

**Authors:** Bingjing Zhao, Jizhen Liu, Xiaoyan Li, Changkui Liu, Jianbo Gao, Min Hu

**Affiliations:** 1 Department of Stomatology, The First Dongguan Affiliated Hospital, Guangdong Medical University, Dongguan, Guangdong China; 2 Scientific Research Platform, The Second Clinical Medical College, Guangdong Medical University, Dongguan, Guangdong China; 3 Laboratory Animal Center, Guangdong Medical University, Dongguan, Guangdong China; 4 Department of Oral and Maxillofacial Surgery, School of Stomatology, Xi’an Medical University, Xi’an, Shanxi China; 5 Centre of Excellence for Advanced Materials, Dongguan, Guangdong China; 6 Department of Stomatology, General Hospital of the PLA, Beijing, China

**Keywords:** Root-analogue implant, Residual stress, 3D printing, Finite element analysis

## Abstract

**Background:**

This study compares the residual stress distribution and stress-release deformation of root-analogue implants (RAIs) and traditional threaded cylindrical implants to assess their clinical applicability and optimization.

**Methods:**

Three implant types (solid RAIs, mesh RAIs, and traditional threaded implants) were analyzed using contour cutting and finite element analysis to evaluate stress distribution and deformation trends after stress release.

**Results:**

RAIs exhibited lower tensile and compressive stresses than threaded implants (T1: 26 MPa vs. 48 MPa, T2: 18 MPa vs. 74 MPa), but showed greater displacement (T1: 0.02 mm, T2: 0.012 mm, T3: 0.0025 mm), mainly in the apical region.

**Conclusions:**

RAIs offer advantages in stress distribution but require further optimization to address deformation. Future research should focus on improving material selection and manufacturing processes to enhance implant stability and long-term success.

**Clinical trial number:**

Not applicable.

## Background

The RAI is designed based on the shape of the missing tooth’s root. The shape of the implant matches the root of the extracted tooth or the shape of the extraction socket, making it suitable for immediate implantation in cases where teeth cannot be preserved due to trauma, decay, or periapical inflammation, but where alveolar bone resorption is not severe [1]. With the application of 3D printing technology, RAIs have gained attention. Compared to traditional tapered or cylindrical implants, RAIs offer the following advantages [2–4]: (1) They fit more precisely into the extraction socket; (2) They do not require gradual drilling of the implant site, allowing for immediate placement after minimally invasive tooth extraction. This simplifies the surgical procedure, reduces costs, and enhances patient comfort; (3) They preserve the maximum amount of alveolar bone, and the stress distribution around the implant is more physiologically appropriate. With the advancement of 3D printing technology, the fabrication of RAIs is no longer a challenge [5–8]. From design to manufacturing, full personalization can be achieved by using the patient’s CT data to design the implant with computer-aided design and then producing it using 3D printing technology. This eliminates complex clinical procedures and offers promising application prospects and value. During implantation, the RAI implant is initially seated into position through finger pressure, followed by gentle tapping with a mallet for final placement. Its primary stability is achieved through precise morphological matching between the implant and the extraction socket, which is clinically verified via palpation and percussion [8].Despite technological advancements in the design and manufacturing of personalized RAIs, their clinical application still faces potential challenges related to stress release and deformation. Current research on these issues remains limited, particularly lacking systematic comparative studies on residual stress distribution and stress-release deformation between RAIs and traditional threaded implants.

Residual stress, as an unavoidable physical phenomenon during material processing, refers to the state of equilibrium stress within a material in the absence of external loads [9]. During additive manufacturing, due to the layer-by-layer accumulation of material and the rapid heating and cooling effects of the laser heat source on the material, a complex residual stress field is generated within the product. These residual stresses not only affect the dimensional accuracy and mechanical properties of the part but may also cause deformation, cracks, or even fractures during use [10–12]. For RAIs, if unremoved residual stress exists internally, once implanted in the body, these stresses may gradually be released as the oral environment changes and masticatory forces continue to act. This release could lead to deformation of the implant [13]. Such deformation not only affects the stability and functional recovery of the implant but may also mechanically stimulate the surrounding bone tissue [14], triggering inflammatory responses, causing bone resorption, and leading to other complications, which ultimately reduce the long-term success rate of the implant. Therefore, in the design and manufacturing process of RAIs, it is crucial to control and eliminate residual stress, which is of great significance for improving the quality and safety of RAIs.

To eliminate residual stress in RAIs, conventional post-processing techniques have been introduced [15]. However, uncertainty remains as to whether these processes can ensure the implants meet the standards required for implantation. Although existing research indicates that certain post-processing techniques significantly alleviate residual stress, the quantification and evaluation of their specific effects still require more supporting evidence. Therefore, it is necessary to conduct comprehensive assessments of the residual stress in RAIs through scientific experimental methods and rigorous data analysis, as well as to explore more effective post-processing techniques to ensure the safety and reliability of the implants.

This study systematically conducted post-processing on two types of personalized RAIs (solid and mesh structures) to evaluate their residual stress states. By analyzing implant deformation, residual stress distribution, and stress-release deformation trends, the results were compared with traditional tapered threaded implants. The objective was to provide a comparative analysis of residual stress distribution and stress-release deformation between RAIs and traditional threaded implants, offering theoretical insights for the optimization and clinical application of RAIs.

## Methods

### Implants (RAIs) design

Import the maxillary bone data into Mimics 17.0 software (Materialise NV). Analyze the threshold using Thresholding, then refine the root structure with Edit Mask in 3D and brush tools. After generating the model, separate the root from the crown. Import the root into 3ds Max 2015 (Autodesk), create a 1 mm outward expansion from the root surface to form the abutment, and combine the two using ProBoolean to generate a personalized implant [6]. Finally, import the model into 3-Matic 11.0 (Materialise NV), select the Porosity Analysis function, choose the porous structure in Entities, specify the pre-porous solid region in Sample Region, and click Apply to calculate and obtain the porosity data of the model.

### Specifications of the samples

**Fig. 1:**
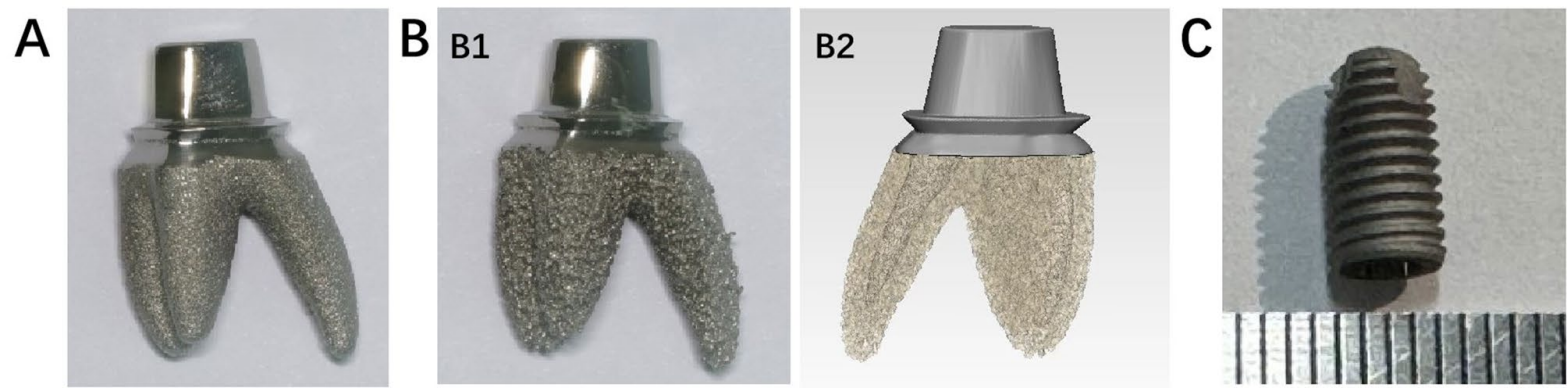
Implant samples: (**A**) RAI (Solid); (**B1**)RAI (Mesh); (**B2**) Corresponding drawings; (**C**) Threaded Implant

The sample consists of three groups of implants, with five specimens in each group, including two root-analog implants (RAIs) and one conventional implant. All implants are fabricated with Ti-6Al-4 V alloy, which has an elastic modulus of 114 GPa and a Poisson’s ratio of 0.3 [16]. T1 and T2 are RAIs (Fig. 1A, B); T1 has a solid structure with a relatively smooth surface, while T2 has a porous structure with a mesh surface. Two-thirds of the root diameter is solid, while the remaining one-third consists of a mesh structure with a porosity of 40-60% and a pore size of 0.2–0.4 mm (Figure 1B2). The RAIs were produced using the EOS M290 (Germany) with a printing power of 280 W and a printing speed of 1200 mm/s. The average diameter of the metal particles was 45–100 μm (Arcam, Sweden, Table 1). After 3D printing, the samples were heated at 650 °C for 2 h, followed by cooling in the furnace for 15 h until reaching room temperature. This process helps relieve internal stress, minimize undesirable phase transformations, optimize grain structure, enhance mechanical properties, and improve surface quality and corrosion resistance [17]. After removal, the samples underwent wire cutting, machining, sandblasting, and polishing. Subsequently, they were cleaned by immersing them in a solution of purified water and a specialized cleaning agent (Xingda Xin, Beijing, China) for ten minutes. After brushing with a brush, they were further cleaned in an ultrasonic cleaning machine for 20 min. Finally, the products were removed for drying. T3 represents conventional implants with basic dimensions of φ4.6 × 10 mm. (Fig. 1C).

**Table 1:** Content of elements in powder for implants

Methods	Al	V	C	Fe	O	*N*	H	Ti
SLM	5.5–6.7	3.5–4.5	<0.08	<0.3	<0.2	<0.05	<0.015	Balance

### Measurement methods and procedures for implants

**Fig. 2:**
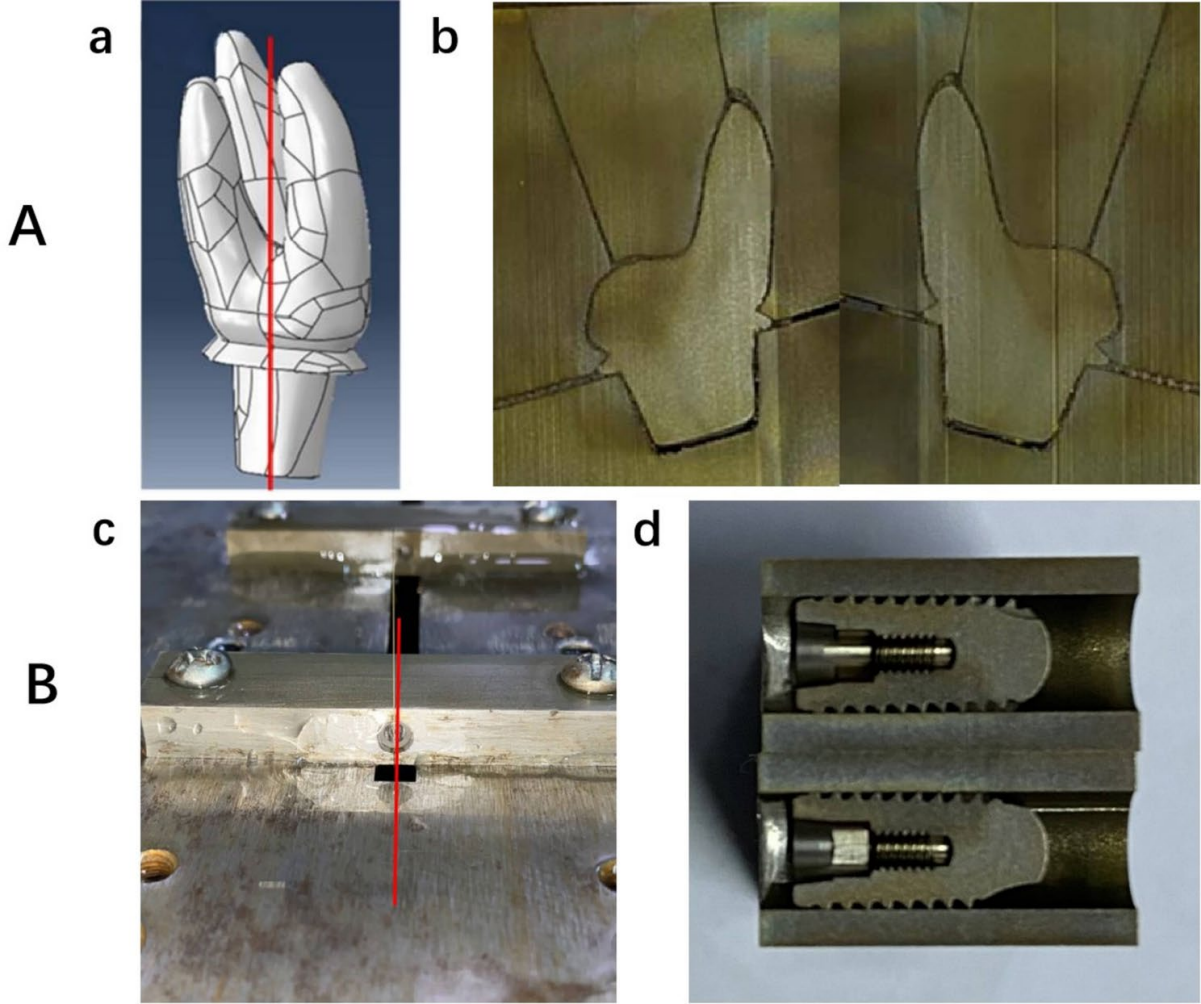
(**A**) RAI: (a) Contour cutting position(The model was developed based on scanned data from the RAI and used for finite element analysis.); (b) Cut surface after cutting. (**B**) Threaded implant: (a) Contour cutting position; (b) Cut surface after cutting

**Fig. 3:**
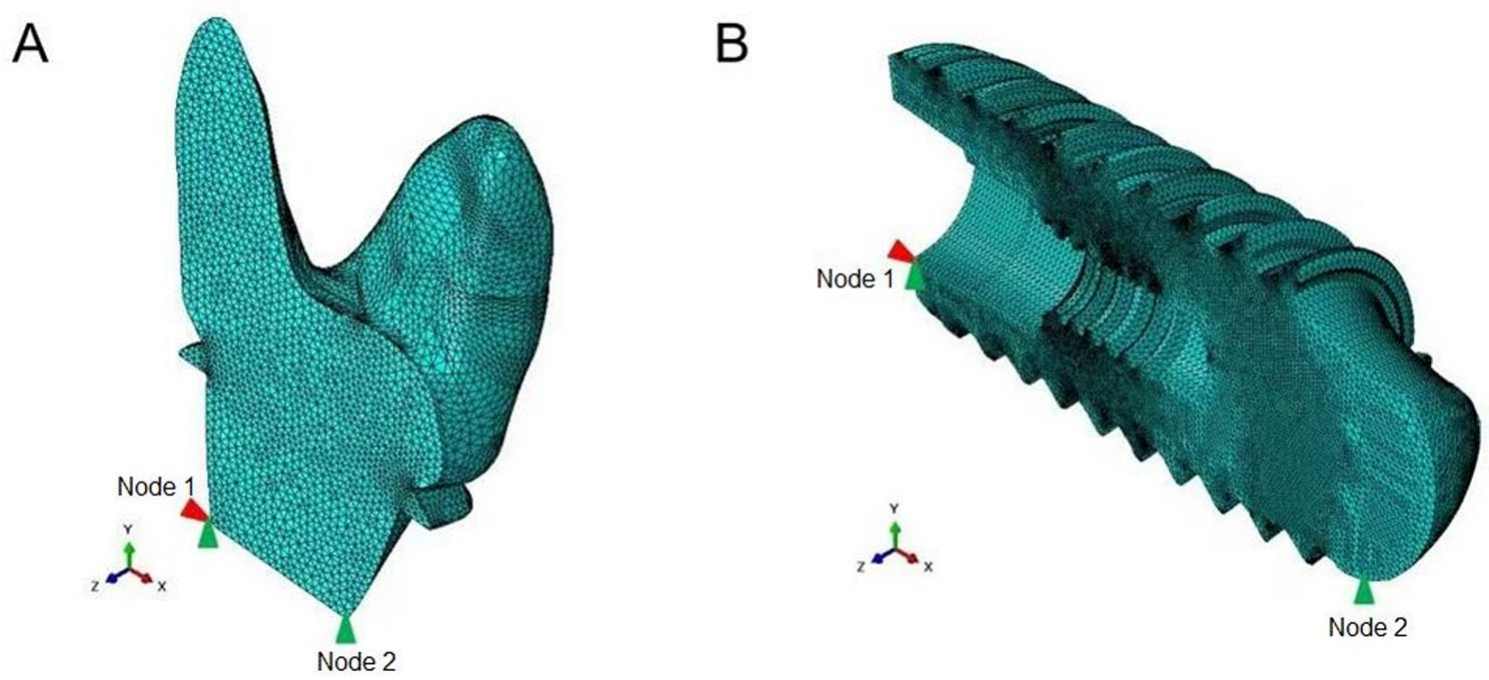
Finite element mesh models. the figure illustrates the finite element mesh representations of different implants used in the study to simulate residual stresses.(**A**) RAI; (**B**) Threaded Implant. Cinematic constraints were applied: Node 1 was fixed in the X and Y translational directions, and Node 2 was constrained along the Y-axis. Red are constrained in the x-direction, Green are constrained in the y-direction)

Residual stress in the normal direction of the implant’s cross-section was measured using the contour method (T/CISA 063-2020). A slow-wire electrical discharge machining (EDM) machine (Sodick AL600P, Sodick, Japan) was used to cut the implant samples into two parts (Fig. 2). The slow-wire cutting machine performed contour cutting along the predetermined cross-section, with cutting conditions set to deionized water at a temperature of 22–24 °C and using a soft copper wire with a diameter of φ = 0.07 mm. After cutting, the two cut surfaces were cleaned without affecting the surface profile, and the surface deformation of the cut faces was measured using an Alicona G5+ (Alicona, Austria), resulting in a cloud of contour points. The measurement point spacing was 0.007 mm. The contour point clouds from both sides were then mirrored, aligned, averaged, and subjected to outlier removal and noise smoothing, resulting in a final two-dimensional point cloud representing the cross-section profile. After obtaining the contour data of the cutting plane, finite element modeling of the sample is performed for stress calculation (Abaqus 2018, ABAQUS, France; Matlab R2019a, MathWorks, USA). The detailed steps are as follows:

A finite element model was developed using second-order C3D10 tetrahedral elements (Fig. 3). The material’s constitutive parameters were defined, and the simulation was performed using a linear perturbation analysis. To prevent rigid-body motion, kinematic constraints were applied: Node 1 was fixed in the X and Y translational directions, and Node 2 was constrained along the Y-axis.Displacement boundary conditions, derived from filtered contour measurements, were imposed onto the resection surface through reverse loading using an inverse algorithm implemented in MATLAB. Specifically, displacement constraints along the Z-axis were mapped from the measured topographical coordinates to their corresponding surface nodes.

**Fig. 4:**
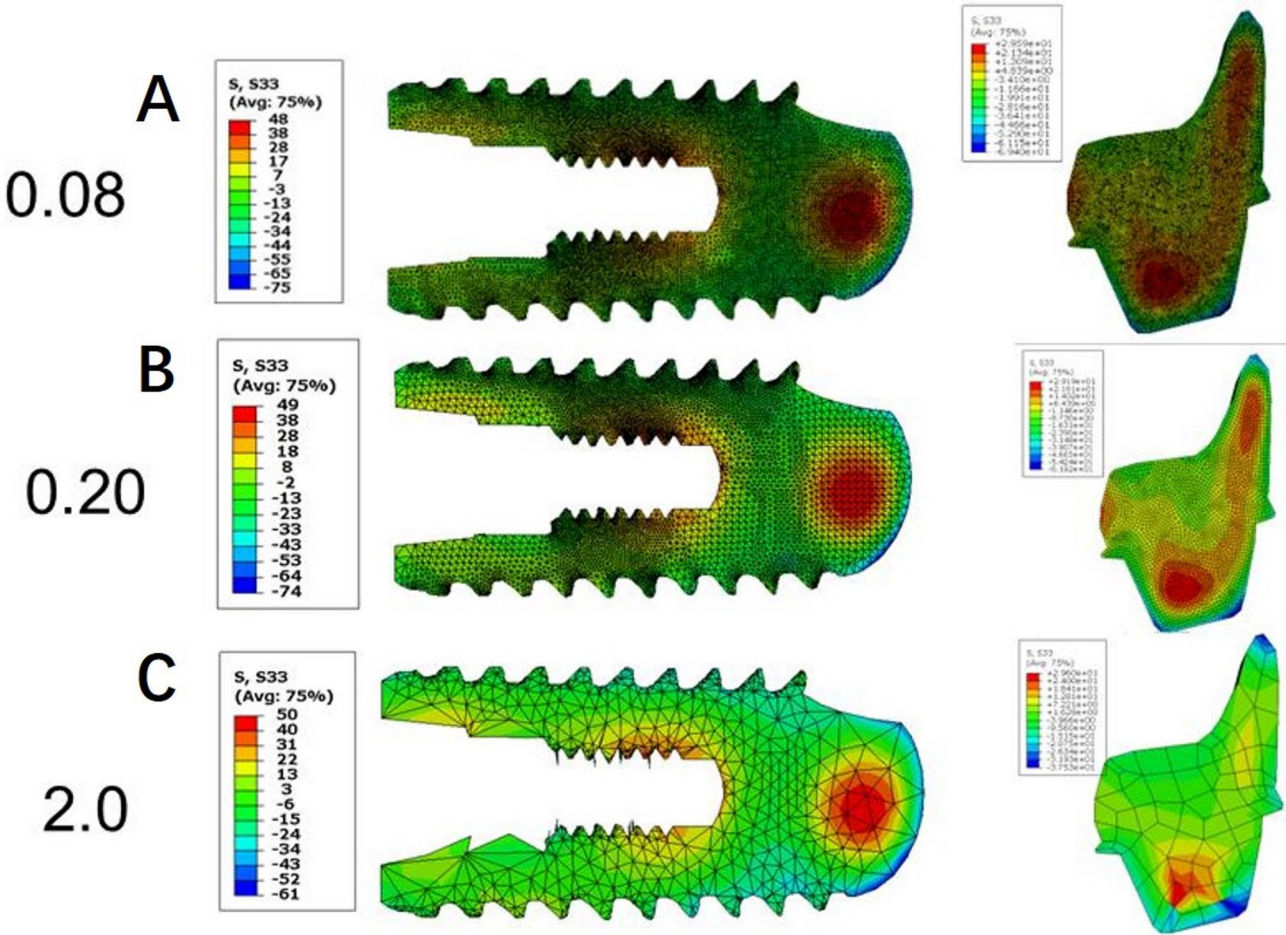
Convergence test on different mesh sizes for the threaded implant and RAI. Mesh sizes of 0.1 mm for the conventional implant and 0.3 mm for the RAI were selected for subsequent contour method calculations.Mesh sizes: (**A**) 0.08 mm; (**B**) 0.2 mm; (**C**) 2.0 mm

Linear elastic simulations were conducted, and the resulting residual stress fields were evaluated and visualized using contour plots. A convergence analysis for mesh sizes ranging from 0.08 mm to 2.0 mm confirmed that convergence was achieved at element sizes below 0.3 mm(Figure 4; Table 2). Consequently, mesh sizes of 0.1 mm for the conventional implant and 0.3 mm for the RAI were selected for subsequent contour method calculations.The data analysis procedure closely followed the guidelines outlined in reference [18, 19].

**Table 2:** Result summary of convergence test on different mesh sizes

	Threaded implant		RAI	
Mesh size(mm)	Stress Max(MPa)	Stress Min(MPa)	Stress Max(MPa)	Stress Min(MPa)
0.08	48	-75	30	-69
0.09	48	-74	30	-69
0.1	48	-74	30	-69
0.12	48	-74	30	-68
0.2	49	-74	29	-67
0.3	49	-73	29	-67
0.4	50	-69	29	-52
1.0	48	-69	31	-40
2.0	50	-61	30	-38

**Fig. 5:**
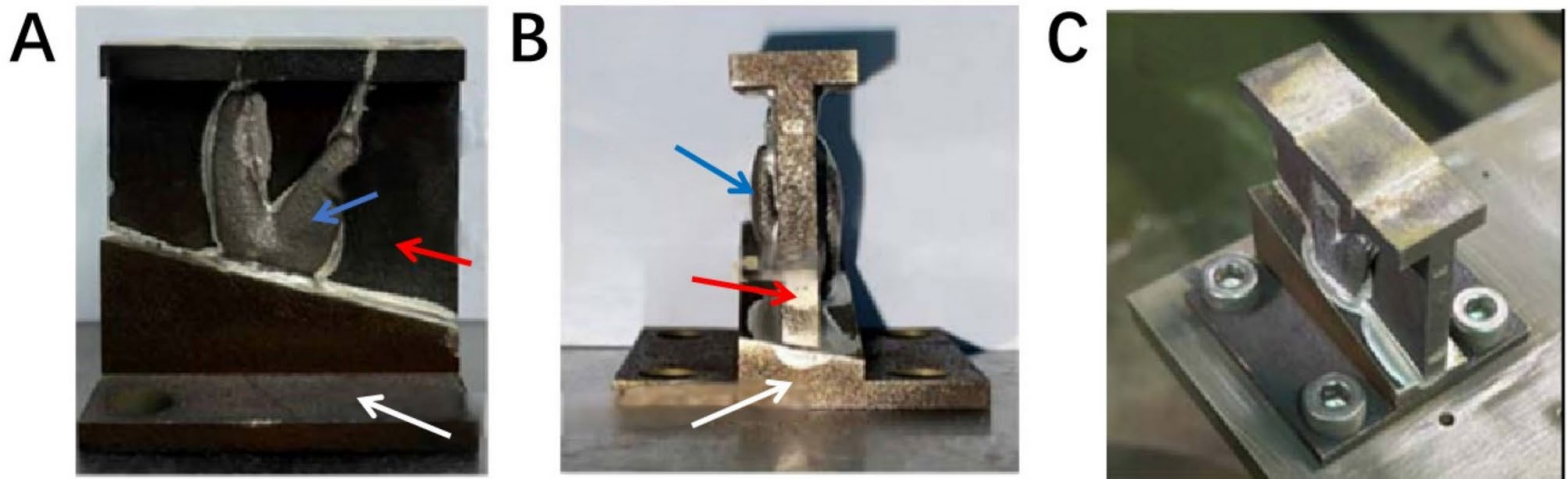
Utilize metal powder laser 3D printing to produce compensatory materials that match the sample’s outer contour, ensuring a stable EDM wire contact area and reducing cutting errors. Custom fixtures secure the sample on the EDM machine, maintaining consistent contact length and minimizing defects from size variations or wire entry/exit, ensuring high-quality measurements: (**A**)(**B**) Assembly display of samples, compensatory material, and fixed fixture; (**C**) Sample fixed on the cutting machine table before cutting. (Blue arrow: RAI; Red arrow: Compensatory material; White arrow: Fixed fixture.)

For RAIs, a compensatory material was designed and produced using a metal powder laser 3D printing device to match the external contour morphology of the samples effectively (Fig. 5). This compensatory material ensures a constant contact area between the EDM metal wire and the sample during the cutting process, reducing cutting errors caused by variations in the cutting section or wire entry and exit. Additionally, a special fixture was designed and fabricated using the metal powder laser 3D printing device (Fig. 5A, B) to secure the specimen on the slow-wire cutting machine’s worktable (Fig. 5C). This setup ensures that the contact length between the slow wire and the workpiece does not change due to differences in cutting position, minimizing cutting defects caused by variations in cutting surface dimensions and wire feed/insertion, ultimately yielding high-quality measurement results.

**Fig. 6:**
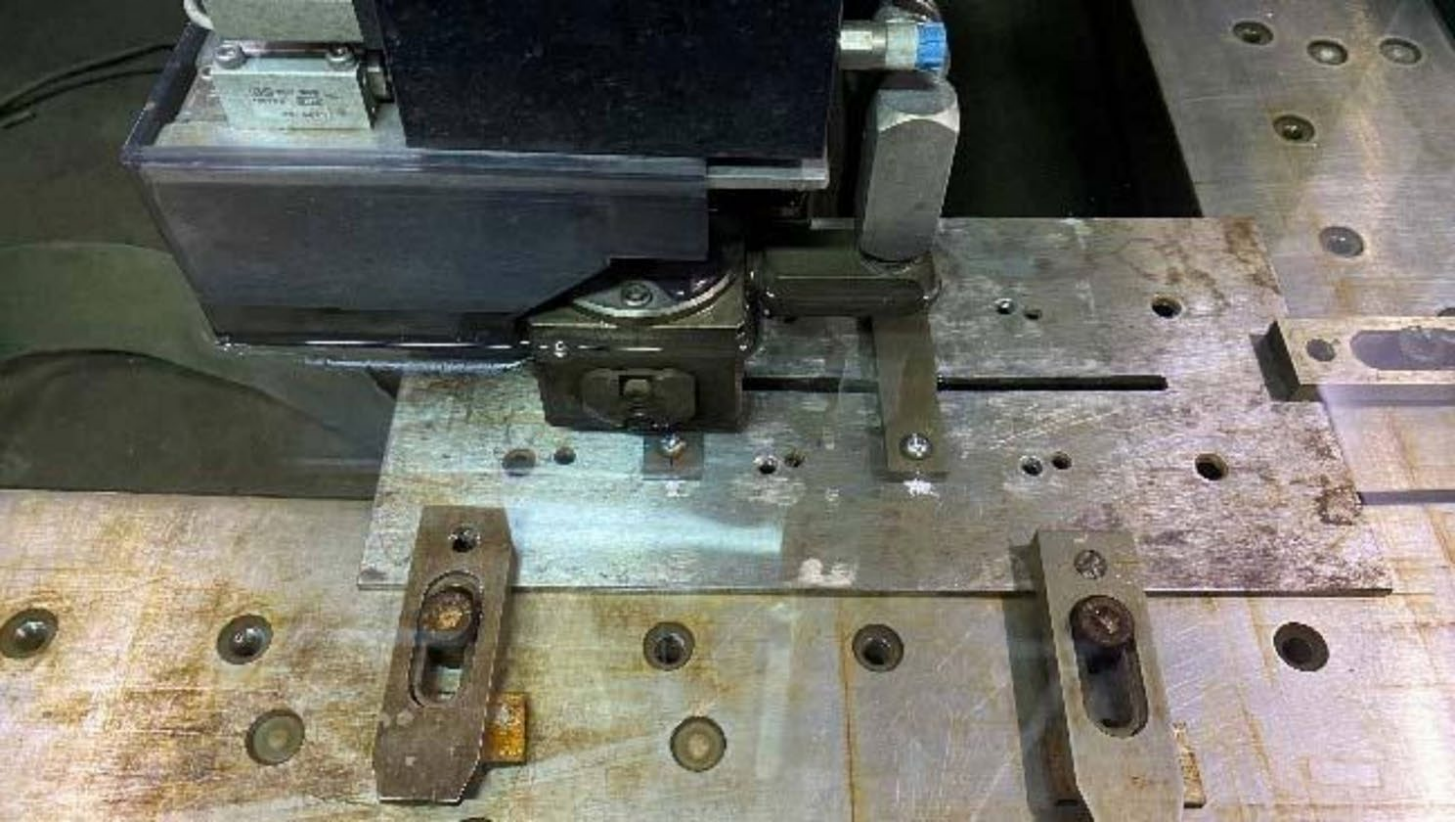
Assembled sample fixed on the cutting machine table: precisely assembled custom fixtures, sacrificial materials, and samples, securing them on the wire-cut EDM machine to maintain stable contact length, reduce cutting defects, and achieve high-quality measurement results

For the threaded implants, placing the threaded samples into a cylindrical sleeve can reduce cutting errors caused by variations in the cutting section or wire entry and exit. As shown in Fig. 6, a precise assembly of the special fixture, sacrificial material, and samples was performed to secure the specimen on the slow-wire cutting machine’s work table. This setup ensures that the contact length between the slow wire and the workpiece does not change due to differences in cutting position, minimizing cutting defects caused by variations in cutting surface dimensions and wire feed/insertion, ultimately achieving high-quality measurement results.

### Statistical analysis

The experimental results were analyzed by SAS 8.0 (SAS Institute Inc., Cary, NC, USA). Normally distributed data were presented as mean ± SD and were compared between groups using one-way ANOVA and Tukey multiple-comparison post hoc test. Non-normally distributed data were described as median (range) and were analyzed using the Kruskal-Wallis test and Dunn test. A P value < 0.05 was considered to indicate a statistically significant difference.

## Results

### Deformation analysis

**Fig. 7:**
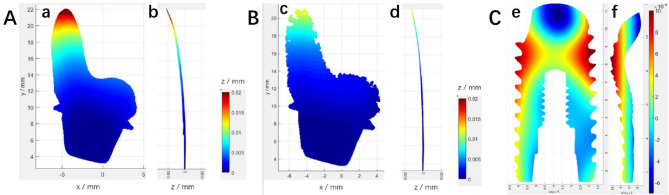
(**A**) T1 sample cut surface contour cloud diagram: (a) Z Perspective; (b) X Perspective; (**B**) T2 sample cut surface contour cloud diagram: (c) Z Perspective; (d) X Perspective; (**C**) T3 sample cut surface contour cloud diagram: (e) Z Perspective; (f) X Perspective

The contour cloud diagram of the cut surface shows that the colors represent the deformation amount in the direction perpendicular to the cut surface. Using the blue area at the bottom as a reference, it can be observed that the deformation at the top of the T1 sample was the largest, approximately 0.02 mm. In comparison, the deformation of T2 was slightly smaller, around 0.012 mm (Fig. 7). The deformation of the T3 sample’s cut surface exhibits an axisymmetric distribution, with the highest negative deformation of -0.0012 mm located at the top of the sample. The deformation gradually diverges from the inside to the surrounding outside, with an overall maximum deformation of 0.0025 mm. The deformation of the T3 sample is less than that of T1 and T2. There are significant differences in the deformation across all three implants (*P* < 0.05). The deformation between T1 and T2, T1 and T3, as well as T2 and T3 implants, all show significant differences (*P* < 0.05).

### Residual stress distribution

**Fig. 8:**
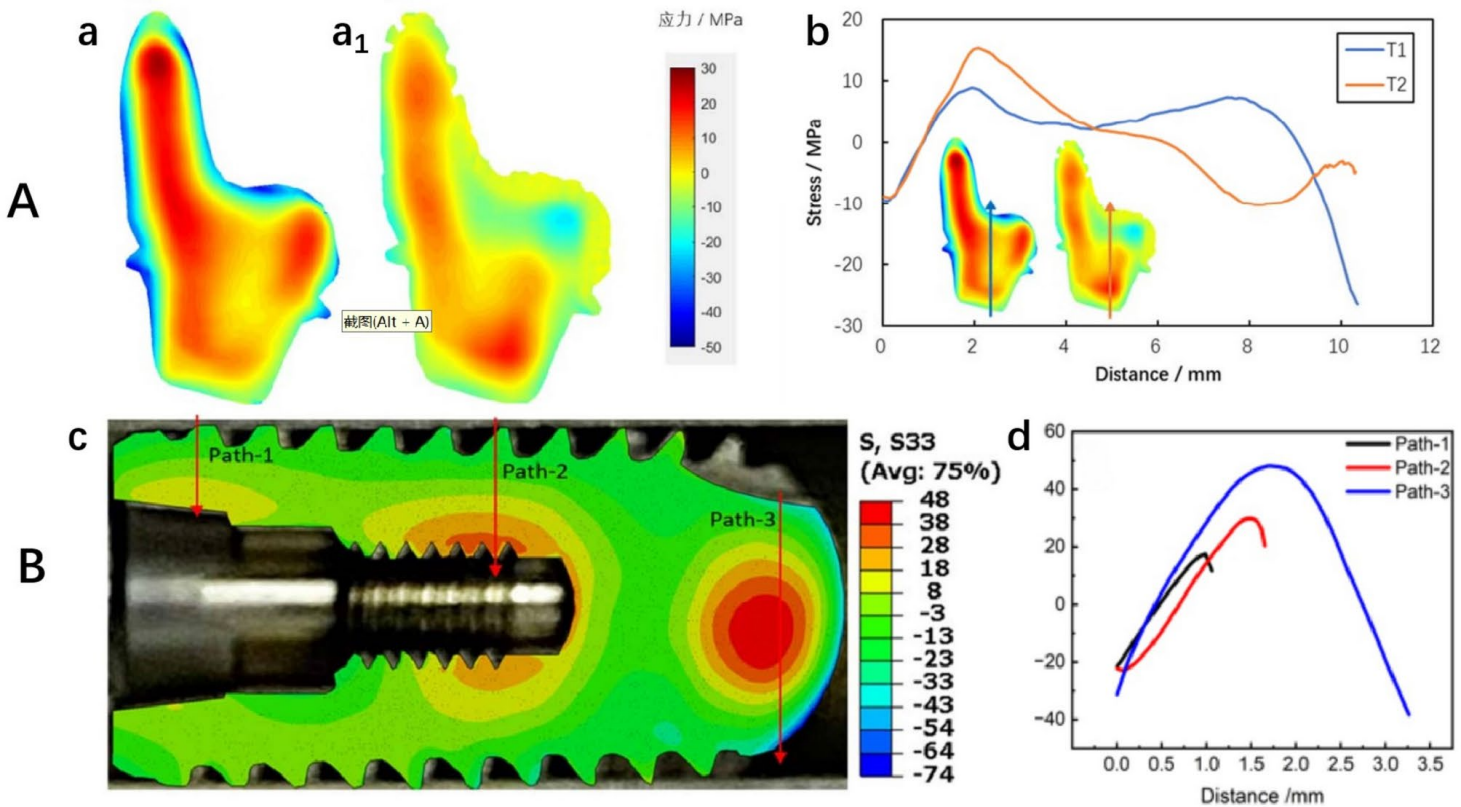
(**A**) RAIs: two-dimensional distribution of normal residual stress of the cut surface: (a) Sample T1; (a1) Sample T2; (b) Stress line distribution at the midpoint between the two samples, with paths indicated by the blue and orange arrows. (**B**) Threaded implant: (c) Two-dimensional distribution of normal residual stress of the cut surface for sample T3; (d) Comparison of stress extraction along different paths: Path-1 and Path-2 show that the stress changes from compressive stress at the surface to tensile stress at the center. Path 3 indicates that the stress at the center of the solid region at the top is tensile, while the surface on both sides exhibits compressive stress. (Colors represent stress magnitude: + indicates tensile stress, - indicates compressive stress.)

All three samples exhibited a two-dimensional distribution of internal tensile stress and external edge compressive stress. The maximum internal tensile stress for T1 was 26 MPa, while the maximum external edge compressive stress was − 47 MPa. For T2, the peak tensile stress was 18 MPa, and the peak compressive stress was − 22 MPa. The maximum internal tensile stress for T3 was 48 MPa, with the maximum compressive stress at -74 MPa (Fig. 8). T2 showed the lowest stress, while T3 exhibited the highest stress, which is inconsistent with the deformation results. There are significant differences in both tensile and compressive stresses across the three implants (*P* < 0.05). Significant differences in tensile and compressive stresses are observed between T1 and T2, T1 and T3, as well as T2 and T3 implants (*P* < 0.05).

### Prediction of stress release deformation

**Fig. 9:**
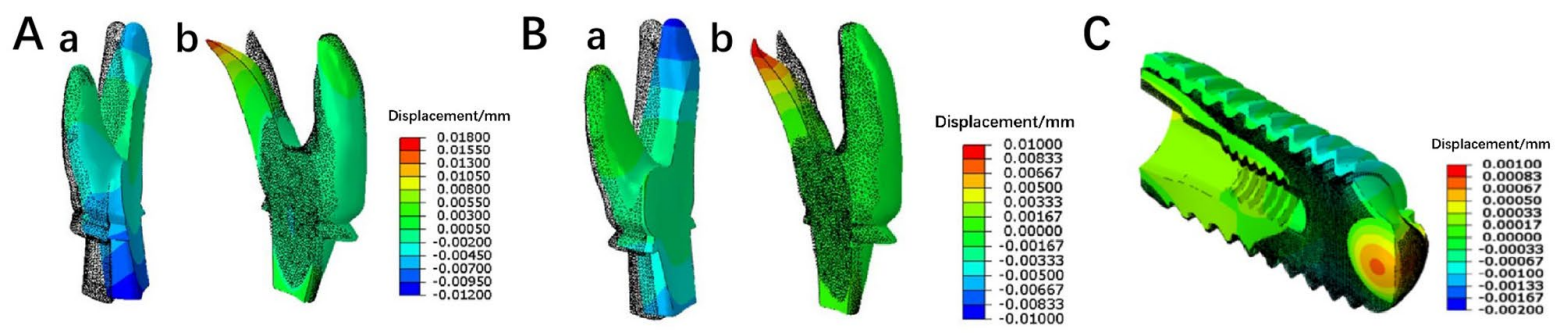
Overall deformation in the direction normal to the cut surface after cutting. The black dot matrix profile represents the position before cutting, while the colored solid model indicates the deformed position after cutting: (**A**) Sample T1: (a) Left side; (b) Right side. (Displacement magnified 200 times.) (**B**) Sample T2: (a) Left side; (b) Right side. (**C**) Sample T3 (Displacement magnified 400 times.)

After cutting, all three samples exhibited deformation in the direction normal to the cut surface. From the deformation results, it can be predicted that the three roots of the RAI will experience displacements that move closer together after stress release. Comparing T1 and T2, it is evident that the overall deformation caused by stress release in T1 is greater than that in T2. The threaded implant expands outward, with displacements smaller than those of the RAIs, which is consistent with the deformation results (Fig. 9).

## Discussion

### Comparative analysis of stress

The experimental results show that the tensile and compressive stresses in the RAIs (T1 and T2) are significantly lower than those in the threaded implant (T3). This finding has important clinical implications. Excessive stress may lead to internal material fatigue or even fracture of the implant, and it could adversely affect the surrounding bone tissue, potentially causing bone resorption or stress shielding phenomena [20, 21]. In addition to directly reducing the risk of fatigue in the internal material of the implant and the potential pressure on the surrounding bone tissue, the lower tensile and compressive stresses in RAIs may also bring other benefits. For instance, lower stress levels may allow the implant to achieve biomechanical stability more quickly after implantation, thereby reducing postoperative recovery time and patient discomfort. Furthermore, a low-stress state may facilitate the process of osseointegration between the implant and bone tissue, as appropriate stress stimulation is one of the key factors for bone cell proliferation and differentiation [22]. This requires further research for confirmation.

### Differences in displacement and optimization recommendations

The experiment revealed that the displacement in RAI is significantly greater than that in threaded implants, with the displacement primarily concentrated in the apical region. This phenomenon may be attributed to two factors: first, the apical region of the RAI is relatively slender, leading to lower mechanical strength and greater susceptibility to deformation; second, the root shape of the RAI is often complex and irregular, which also increases the risk of deformation during the printing process [23].

To optimize this issue, future designs could consider reducing the apical region without compromising implantation and initial stability. Additionally, designing irregular roots into more regular shapes could help lower the risk of deformation. Furthermore, improvements in material selection and processing techniques can be explored. For example, using materials with higher strength and better deformation resistance could significantly enhance the overall stability of the implants. It is also important to optimize temperature control, cutting parameters, and heat treatment processes during manufacturing to minimize the stresses and deformations introduced during processing [24].

### Prediction of stress release deformation and bone tissue remodeling

The stress-release deformation prediction results indicate that the root region of the personalized implants experiences greater displacement, with the roots tending to move closer to each other. This deformation trend could have complex effects on the surrounding bone tissue. Based on the observed deformation characteristics, the following clinical scenarios may occur.

### Bone tissue adaptation and remodeling

If the surrounding bone tissue can adapt to the implant’s deformation and undergo necessary remodeling, the stability of the implant may remain unaffected. Bone remodeling and resorption can effectively distribute the stresses generated by the implant, ensuring long-term stability and functional recovery. In this case, the implant may achieve strong osseointegration with the bone tissue, further enhancing stability.

### Delayed bone tissue remodeling

However, if the bone tissue remodeling process lags behind the speed of implant deformation, stress concentration may occur. This is particularly true when the implant undergoes significant displacement, potentially creating localized stress concentration areas between the bone tissue and the implant. Such stress concentrations could lead to adverse outcomes like bone resorption [25]. The inability of the bone tissue to adequately adapt to the implant’s deformation may slow down and reduce the quality of osseointegration, ultimately affecting the implant’s long-term stability. To improve long-term stability, future research should focus on optimizing design and material selection to reduce implant deformation and promote synchronized bone remodeling, ensuring successful osseointegration and implant stability.

In conclusion, the dynamic relationship between implant deformation and bone tissue remodeling requires further investigation, especially in the context of personalized implants, to achieve better clinical outcomes and long-term success.

### Advantages of porous implants

According to the mechanical homeostasis theory proposed by Frost [26], Bone remodeling is determined by the applied strain levels. This theory divides strain into five primary ranges and correspondingly defines bone remodeling. Among them, εoct represents the octahedral shear (equivalent) strain, which is considered the most suitable strain for the mechanical homeostasis theory. When bone is overloaded, the accumulation of sustained damage can override its adaptive and self-repairing capabilities. In the adaptation model, this manifests as a rapid decline in bone stiffness and/or mass once a certain overload threshold is exceeded. Moreover, lower trabecular bone density leads to higher strain within the bone [27–29].

Although traditional titanium (Ti) implants exhibit excellent biomechanical properties, their stiffness is significantly higher than that of natural bone, making them prone to stress shielding, aseptic loosening, and bone resorption [30]. To address these drawbacks, porous titanium has been introduced to reduce implant stiffness and mimic bone properties, thereby better matching the mechanical behavior of natural bone [31]. In recent years, the rapid development of additive manufacturing (3D printing) technology has made it possible to fabricate low-stiffness porous titanium implants. Unlike surface-porous structures, the unique interconnected porous structure of porous Ti-6Al-4 V alloy not only promotes cell adhesion, proliferation, and nutrient transport [32] but also effectively mitigates stress shielding [33]. Additionally, its mechanical properties are highly tunable, allowing precise adjustments to meet various clinical needs [29], significantly enhancing bone ingrowth [34], and thereby improving the long-term stability of implants. Numerous in vivo studies have confirmed that 3D-printed porous titanium implants can significantly enhance osteogenic responses [35–37], providing a superior solution for bone repair and regeneration.

Therefore, porous Ti-6Al-4 V alloy demonstrates outstanding performance in cytocompatibility, mechanical properties, and osseointegration, making it widely regarded as one of the most promising biomimetic implant materials available today.

In the comparison between the two RAIs, the porous implant (T2) demonstrated lower residual stress, indicating that it is more suitable for implantation. This result suggests that the design of the porous structure has a distinct advantage in reducing residual stress, which may be related to its larger surface area and more uniform stress distribution. Additionally, the porous structure may help disperse stress within the implant, further lowering local stress peaks [38, 39]. These advantages position porous implants for broader development prospects in clinical applications. However, to ensure their widespread use in clinical practice, several key issues need to be addressed. For instance, how can the integrity and stability of the porous structure be maintained to withstand long-term occlusal forces and other oral environmental impacts? How can the dimensions and shapes of the porous structure be optimized to better accommodate the oral conditions and needs of different patients? Through interdisciplinary collaboration and the application of innovative technologies, it is believed that the potential advantages of porous implants can be gradually unveiled, facilitating the advancement of their clinical applications.

Based on the above analysis, considering the stress and deformation characteristics, the decision to use personalized RAIs instead of traditional threaded implants typically involves the following factors:

### Bone tissue condition and structure

When selecting an implant, it is essential to consider the patient’s bone tissue condition and structural characteristics [28, 29]. For patients with a faster bone remodeling rate (such as younger individuals with higher bone density and stronger bone remodeling ability), even though RAI may cause larger displacements and stress concentrations if the bone tissue can adapt to these changes and remodel accordingly, the implant’s long-term stability and functional recovery may be improved. Additionally, in immediate molar implants, where molars are multi-rooted and the extraction socket significantly differs from the shape of threaded implants, bone grafting, and implant embedding are often required to enhance stability. RAI, however, closely matches the shape of the extraction socket, providing better defect filling, reducing bone tissue damage, preserving bone structure, and lowering the need for bone grafting, offering distinct advantages in complex alveolar conditions.

### Masticatory force and stress distribution

Young patients typically exhibit higher masticatory forces, especially in immediate molar implants, where the transmission characteristics of these forces must be considered. Traditional threaded implants, with their single-column design, are prone to stress concentration at the implant neck under high occlusal forces, potentially leading to bone resorption and implant instability. In contrast, RAI, with a shape resembling that of natural molars, provides closer contact between the surface and bone tissue. This helps distribute the masticatory forces more evenly, reducing stress concentration at the neck, lowering the risk of bone resorption, and better adapting to strong occlusal forces, thus protecting the integrity of the surrounding bone [40].

Therefore, RAI may be more suitable for younger patients, particularly those with good bone density and higher occlusal forces. RAI can better distribute masticatory forces, reduce stress concentration at the neck, maintain bone height, and prevent bone resorption caused by excessive stress concentration. Further research is needed to confirm these findings.

### Limitations

The limitations of this study include a small sample size, lack of in vivo experimental validation, and potential variability in the manufacturing process. Future research should aim to expand the sample size and incorporate in vivo experiments to validate the clinical applicability of the findings.

## Conclusions

In conclusion, this study, through systematic experimental design and scientific analysis methods, explored the differences in residual stress distribution and stress release deformation between RAIs and threaded implants. The research findings provide a scientific basis for the clinical selection of suitable implants. Although the RAIs showed significantly lower stress compared to the threaded implants, the larger deformation raises questions about whether bone remodeling can adapt to this deformation. Therefore, future research needs to comprehensively consider aspects such as mechanical properties, bone tissue response, and clinical applicability, while also exploring more effective post-processing techniques and better implant designs. These efforts will provide critical support for the further development and application of RAIs.

## Data Availability

All data generated or analysed during this study are included in this published article.

## Abbreviations

RAI: Root-analogue implant
EDM: Electrical discharge machining
